# Compare the epidemiological and clinical features of imported and local COVID-19 cases in Hainan, China

**DOI:** 10.1186/s40249-020-00755-7

**Published:** 2020-10-19

**Authors:** Biao Wu, Zi-Ying Lei, Kun-Liang Wu, Jian-Rong He, Hui-Juan Cao, Juan Fu, Feng Chen, Yuan Chen, Bao Chen, Xiao-Li Zhou, Tao Huang, Tao Wu, Yong-Guo Du, Suo-Xian Chen, Fu-Rong Xiao, Zhi-Liang Gao, Jing He, Feng Lin, Bing-Liang Lin

**Affiliations:** 1 Department of Infectious Diseases, Hainan General Hospital, Hainan Affiliated Hospital of Hainan Medical University, Haikou, China; 2 Office of Diseases Control and Prevention, Hainan General Hospital, Hainan Affiliated Hospital of Hainan Medical University, Haikou, China; 3 Department of Infectious Diseases, the Third Affiliated Hospital of Sun Yat-Sen University, 600 Tianhe Road, Guangzhou, China; 4 GuangDong Provincial Key Laboratory of Liver Diseases, the Third Affiliated Hospital of Sun Yat-sen University, Guangzhou, China; 5 Department of Infectious Diseases, The Second Affiliated Hospital of Hainan Medical University, Haikou, China; 6 Nuffield Department of Women’s and Reproductive Health, University of Oxford, Oxford, UK; 7 Radiology Department, Hainan General Hospital, Hainan Affiliated Hospital of Hainan Medical University, Haikou, China; 8 Clinical laboratory, Hainan General Hospital, Hainan Affiliated Hospital of Hainan Medical University, Haikou, China; 9 Key Laboratory of Tropical Disease Control (Sun Yat-sen University), Ministry of Education, Guangzhou, China

**Keywords:** Coronavirus disease 2019, Severe acute respiratory syndrome coronavirus 2, Epidemiology, Clinical features, Prevention and control

## Abstract

**Background:**

Effective management of imported cases is an important part of epidemic prevention and control. Hainan Province, China reported 168 coronavirus disease 2019 (COVID-19), including 112 imported cases on February 19, 2020, but successfully contained the epidemic within 1 month. We described the epidemiological and clinical characteristics of COVID-19 in Hainan and compared these features between imported and local cases to provide information for other international epidemic areas.

**Methods:**

We included 91 patients (56 imported and 35 local cases) from two designated hospitals for COVID-19 in Haikou, China, from January 20 to February 19, 2020. Data on the demographic, epidemiological, clinical and laboratory characteristics were extracted from medical records. Patients were followed until April 21, 2020, and the levels of antibodies at the follow-ups were also analysed by the Wilcoxon matched-pairs signed ranks test.

**Results:**

Of the 91 patients, 78 (85.7%) patients were diagnosed within the first three weeks after the first case was identified (Day 1: Jan 22, 2020), while the number of local cases started to increase during the third week. No new cases occurred after Day 29. Fever and cough were two main clinical manifestations. In total, 15 (16.5%) patients were severe, 14 (15.4%) had complicated infections, nine (9.9%) were admitted to the intensive care unit, and three died. The median duration of viral shedding in feces was longer than that in nasopharyngeal swabs (19 days vs 16 days, *P* = 0.007). Compared with local cases, imported cases were older and had a higher incidence of fever and concurrent infections. There was no difference in outcomes between the two groups. IgG was positive in 92.8% patients (77/83) in the follow-up at week 2 after discharge, while 88.4% patients (38/43) had a reduction in IgG levels in the follow-up at week 4 after discharge, and the median level was lower than that in the follow-up at week 2 (10.95 S/Cut Off (S/CO) vs 15.02 S/CO, *P* <  0.001).

**Conclusion:**

Imported cases were more severe than local cases but had similar prognoses. The level of IgG antibodies declined from week 6 to week 8 after onset. The short epidemic period in Hainan suggests that the epidemic could be quickly brought under control if proper timely measures were taken.

## Background

Coronavirus disease 2019 (COVID-19), which is caused by severe acute respiratory syndrome coronavirus 2 (SARS-CoV-2) and is characterized by lung damage, was first reported in December 2019 [1, 2]. By September 7, 2020, there has been reported 27 032 617 cases and 881 464 deaths in 213 countries and territories worldwide and, with a crude mortality of 3.3% [3]. Although COVID-19 is likely a zoonotic disease, it can be transmitted from person to person [4, 5], with a reproductive number of 1.5–4 [6, 7]. COVID-19 is mainly transmitted by contact and droplets but can also be transmitted through the digestive tract and conjunctiva [8, 9]. At present, the numbers of COVID-19 cases in USA, India, Brazil and other countries are increasing rapidly, and many are locally incident cases [3]. As is known, the containment of epidemics at the early stage is the most critical, effective and efficient before the outbreak goes out of control. Although many countries once contained the spread of the epidemic, new imported cases are now emerging again. The prevention and control of the epidemic still faces great challenges.

**Fig. 1:**
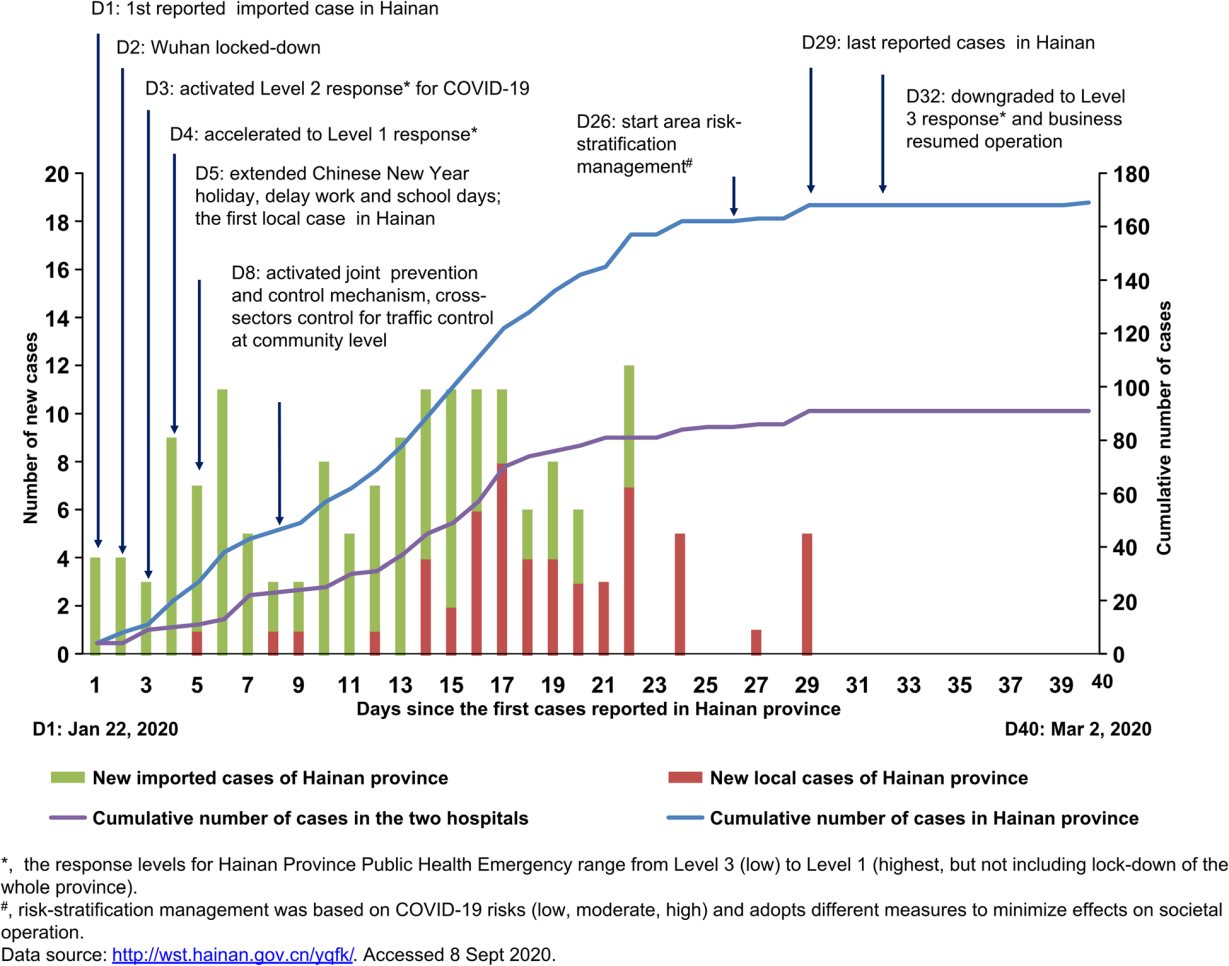
Epidemic tendency of COVID-19 and the measures adopted for epidemic control in Hainan Province. COVID-19: Coronavirus disease 2019

Hainan Province is a popular domestic and international tourist destination with 9.34 million permanent residents. During the Chinese Spring Festival holiday, many people spent their vacations on Hainan Island. According to the statistics, from December 30, 2019, to January 22, 2020, which was the day before Wuhan closed its outbound channel, approximately 74 600 tourists had travelled from Wuhan to Hainan by plane [10], and the first case of COVID-19 in Hainan was reported on January 22, 2020 (Day 1). Compared with other regions except Wuhan, Hainan Province was under greater pressure to prevent and control the epidemic. However, the last confirmed patient in Hainan was reported on February 19, 2020 (Day 29) (Fig. 1) [11]. The relatively short epidemics period is largely attributable to the strict isolation and prevention measures implemented from top to bottom throughout the country, and it also implies that the measures that Hainan adopted to contain the epidemic were timely and successful. Thus, Hainan’s experience may have important implications for other international epidemic areas.

This study is to analyses the epidemiological and clinical characteristics of COVID-19 in two designated hospitals in Haikou, as well as the government’s prevention and control measures, and compares these features between imported and local cases, which may provide an instructive example for countries and regions that are vulnerable to upcoming epidemics.

## Methods

### Study design and participants

We retrospectively included all patients with COVID-19 in Hainan General hospital and the Second Affiliated Hospital of Hainan Medical University from January 22 to February 19, 2020. These two hospitals were designated hospitals for treating adult patients in Haikou, the capital city of Hainan Province. Patients were followed until April 21, 2020, when the last discharged patient had been followed for four weeks. All patients with COVID-19 enrolled in this study were diagnosed according to the WHO interim guidance [12] and were divided into an imported case group and a local case group according to epidemiological data.

### Definitions

Imported cases were defined as the patients who came from a COVID-19 epidemic area within 14 days or from a COVID-19 epidemic area and could not trace the source of infection. The local cases were defined as the patients who stayed in the locality for more than 14 days before onset and had not gone to an epidemic area. If two or more confirmed cases were found concurrently and there was the possibility of human-to-human transmission due to close contact or infection through co-exposure, then the case is determined as a clustered case [13, 14]. Fever was defined as an axillary temperature of at least 37.3 °C. Acute respiratory distress syndrome (ARDS) was defined according to the Berlin definition [15]. We defined the degree of severity of COVID-19 (severe vs mild) at the time of admission using the American Thoracic Society guidelines for community-acquired pneumonia [16].

### Data collection

The epidemiological characteristics (including the residence, whether the patient was from the epidemic area, recent exposure history, etc.), clinical symptoms and signs, laboratory data, chest computed tomography (CT) findings, SARS-CoV-2 RNA, IgM antibody and IgG antibody against SARS-CoV-2 were extracted from electronic medical records.

### Laboratory confirmation of SARS-CoV-2 infection

SARS-CoV-2 RNA testing was performed by the hospital’s laboratory and the key laboratory of Hainan Centre for Disease Control and Prevention (CDC), China, by real-time reverse transcription polymerase chain reaction (RT-PCR) according to the diagnosis protocol for COVID-19 established by the WHO [17]. The nasopharyngeal swab and feces were collected every 2–7 days during hospitalization and twice every seven days during follow-up for discharged cases. Then, the RNA samples from the nasal swab and feces specimens were extracted and subjected to real-time RT-PCR testing using SARS-CoV-2-specific primers and probes. Specifically, the primers for the open reading frame 1ab (ORF1ab) were 5′-CCCTGTGGGTTTTACACTTAA-3′(Forward) and 5′-ACGATTGTGCATCAGCTGA-3′(Reverse), and the corresponding probe was 5′-CY3-CCGTCTGCGGTATGTGGAAAGGTTATGG-BHQ1–3′. Primers for the nucleocapsid protein (N) were 5′-GGGGAACTTCTCCTGCTAGAAT-3′ (Forward) and 5′-CAGACATTTTGCTCTCAAGCTG-3′ (Reverse), and the corresponding probe was 5′-FAM-TTGCTGCTGCTTGACAGATT-TAMRA-3′. The duration of SARS-CoV-2 shedding was defined as the time from symptom onset to the first negative SARS-CoV-2 RNA test after the last SARS-CoV-2 RNA positive test during the follow-up.

### Antibody measurement

IgM and IgG antibodies against SARS-CoV-2 in the plasma samples were tested using the Diagnostic Kit for Novel Coronavirus (2019-nCOV, SARS-CoV-2) IgM/IgG Antibody (Magnetic particle CLIA) supplied by Bioscience (ChongQing, China) Diagnostic Technology Co. according to the manufacturer’s instructions.

### Statistical analysis

Continuous variables were expressed as the mean (standard deviation [*SD*]) or median (interquartile range (IQR)) and compared by the *t*-test or Mann-Whitney U test. Categorical variables were expressed as the frequency (%) and compared by the *χ*^2^ test or Fisher’s exact test between the imported cases and local cases. For the laboratory indicators, we categorized the results as normal or abnormal (increased or decreased). IgG levels during the follow-up were compared using the Wilcoxon matched-pairs signed ranks test. We used SPSS (version 26.0, IBM, New York, USA) for all analyses.

### Ethical statement

This study was approved by the Ethics Committee of Hainan General Hospital and the Second Affiliated Hospital of Hainan Medical University (HN-2020-31), and written informed consent was obtained from all patients.

## Results

### Demographics and epidemiological characteristics

All 91 patients with confirmed COVID-19 in Hainan General Hospital (*n* = 69) and The Second Affiliated Hospital of Hainan Medical university (*n* = 22) were enrolled. Of the 91 patients, 56 were imported and 35 were local patients. The mean age was 50 years, and 57.1% were male. This outbreak lasted one month from the first patient admittance on January 20, 2020, to the last patient on February 19, 2020 (Fig. 1). Of these patients, 78 were admitted within the first 3 weeks after the first case was identified, 88.6% (31/35) of local patients were admitted by day 14, and these statistics were similar to the overall patient statistics in Hainan Province. Of all 168 patients, 142 new cases were confirmed before day 21, and 52 local patients were diagnosed after day 14 (Fig. 1). Among 56 imported patients in two hospitals, 53 (94.6%) came from Wuhan City and its surrounding area. The median interval period between leaving Wuhan to symptom onset in 53 patients was 5 days (IQR: 2–12, range 1–34). Overall, 42 (46.2%) patients had a history of contact with confirmed COVID-19 patients, with a median interval before onset of 8 days (IQR: 4–13, range: 1–22). Compared to imported patients, the local patients were significantly younger (mean age, 46 years vs 52 years; *P* = 0.03), more likely to occur in cluster outbreaks (77.1% vs 46.4%, *P* = 0.004), and had close contact with COVID-19 patients (68.6% vs 32.1%, *P* = 0.001) (Table 1).

**Table 1:** Demographics and epidemiological characteristics of patients with COVID-19

	All patients (***N*** = 91)	Local cases (***n*** = 35)	Imported cases (***n*** = 56)	***P*** values
**Characteristics**				
Age, years, Mean (*SD*)	50 (14)	46 (12)	52 (15)	0.030
Range	21–83	21–73	27–83	
≥ 29	6 (6.6%)	2 (5.7%)	4 (7.1%)	0.410
30–39	20 (22.0%)	10 (28.6%)	10 (17.9%)	
40–49	15 (16.5%)	8 (22.9%)	7 (12.5%)	
50–59	25 (27.5%)	9 (25.7%)	16 (28.6%)	
60–69	19 (20.9%)	5 (14.3%)	14 (25.0%)	
≥ 70	6 (6.6%)	1 (2.9%)	5 (8.9%)	
Sex				0.670
Female	39 (42.9%)	16 (47.2%)	23 (41.1%)	
Male	52 (57.1%)	19 (52.8%)	33 (58.9%)	
Chronic medical illness	31 (34.1%)	8 (22.9%)	23 (41.1%)	0.074
Hypertension	12 (13.2%)	2 (5.7%)	10 (17.9%)	0.120
Cardiovascular disease	5 (5.5%)	1 (2.9%)	4 (7.1%)	0.645
Diabetes	5 (5.5%)	0 (0.0%)	5 (8.9%)	0.152
Respiratory system disease	7 (7.7%)	1 (2.9%)	6 (10.7%)	0.243
Thyroid disease	2 (2.2%)	0 (0.0%)	2 (3.6%)	0.521
Chronic liver disease	5 (5.5%)	1 (2.9%)	4 (7.1%)	0.645
Chronic kidney disease	3 (3.3%)	1 (2.9%)	2 (3.6%)	1.000
Digestive system disease	2 (2.2%)	1 (2.9%)	1 (1.8%)	1.000
Malignant tumor	1 (1.1%)	1 (2.9%)	0 (0.0%)	0.385
Other	6 (6.6%)	1 (2.9%)	5 (8.9%)	0.400
**Epidemiological survey**				
Live or travel history in epidemic area^a^	53 (58.2%)	0 (0.00%)	53 (94.6%)	< 0.001
Time of out of epidemic area to onset, days, *n* = 53, Median (IQR), [range]	5 (2–10) [1–34]	NA	5 (2–10) [1–34]	NA
Close contacts with COVID-19 patient	42 (46.2%)	24 (68.6%)	18 (32.1%)	0.001
Time of contacted COVID-19 patient to onset, days, *n* = 26, Median (IQR) [range]	26/42; 8 (4–13) [1–22]	17/24; 6 (4–15) [1–22]	9/18; 8 (5–16) [2–20]	0.570
Cluster outbreak	53 (58.2%)	27 (77.1%)	26 (46.4%)	0.004

Data are *n* (%) unless specified otherwise. *N* is the total number of patients with available data. *P* values for comparing two groups were derived using Fisher’s exact test for categorized variables and the *t*-test for continuous variables

^a^Epidemic area refers to Wuhan and other epidemic areas in Hubei Province

*COVID-19* Coronavirus disease-19; *ARDS* Acute respiratory distress syndrome; *NA* Not available; *SD* Standard deviation; *IQR* Interquartile range

### Clinical characteristics and complications

The most common symptoms at the onset of illness were fever (79.1%), dry cough (79.1%), expectoration (39.6%), fatigue (38.5%), and shortness of breath (29.7%), while diarrhea (14.3%) and nausea and vomiting (7.7%) were not rare. In total, 87 (95.6%) patients had more than one sign or symptom, and 23 patients had combined fever, cough, and shortness of breath. Only four (4.4%) cases had no symptoms. The median highest temperature was 38.0 °C, and the median duration of fever was eight days. Nine (9.9%) patients were admitted and transferred to the ICU because of the ARDS and organ dysfunction. The median durations from the first symptoms to hospital admission and ARDS were five days (IQR: 3–9) and 8 days (IQR: 6–10), respectively.

Clinically, patients were diagnosed as mild (76 cases, 83.5%) or severe (15 cases, 16.5%) cases. The fifteen severe patients included three local and 12 imported cases. Among these severe patients, 14 had complicated bacterial infections, nine had septic shock, 13 had ARDS, six had multiple organ dysfunction syndrome (MODS), and three patients died. Compared with local patients, imported patients had a higher prevalence of fever (*P* = 0.001), a higher peak temperature (*P* = 0.028), and more complicated infections (*P* = 0.043) and tended to be more severe (21.4% vs 8.6%) (Table 2).

**Table 2:** Clinical characteristics of patients with COVID-19

	All patients (*N* = 91)	Local cases (*n* = 35)	Imported cases (*n* = 56)	*P* values
**Illness station**				
First symptom to, days, Median (IQR)				
Hospital admission	5.0 (3.0–9.0)	6.0 (2.0–10.0)	5.0 (3.0–7.8)	0.670
ARDS	8.0 (5.5–9.5)	*N* = 2, Range 4–6	*N* = 7, 9.0 (6.0–10.0)	0.184
Admission to intensive care unit	9 (9.9%)	2 (5.7%)	7 (12.5%)	0.291
Clinical classification				0.149
Mild	76 (83.5%)	32 (91.4%)	44 (78.6%)	
Severe	15 (16.5%)	3 (8.6%)	12 (21.4%)	
**Signs and symptoms**				
Fever	72 (79.1%)	21 (60.0%)	51 (91.1%)	0.001
Peak temperature, °C, Median (IQR)	38.0 (37.5–38.7)	37.8 (36.9–38.6)	38.0 (37.7–38.7)	0.028
Days of fever, Median (IQR)	8.0 (4.0–10.0)	6.0 (3.0–10.5)	8.0 (4.8–10.0)	0.506
Dry cough	72 (79.1%)	30 (85.7%)	42 (75.0%)	0.221
Expectoration	36 (39.6%)	16 (45.7%)	20 (35.7%)	0.343
Fatigue	35 (38.5%)	11 (31.4%)	24 (42.9%)	0.276
Shortness of breath	27 (29.7%)	9 (25.7%)	18 (32.1%)	0.639
Myalgia	11 (12.1%)	3 (8.6%)	8 (14.3%)	0.521
Diarrhea	13 (14.3%)	3 (8.6%)	10 (17.9%)	0.218
Sore throat	10 (11.0%)	2 (5.7%)	8 (14.3%)	0.306
Nausea and vomiting	7 (7.7%)	2 (5.7%)	5 (8.9%)	0.703
More than one sign or symptom	87 (95.6%)	33 (94.3%)	54 (96.4%)	0.637
Fever, cough, and shortness of breath	23 (25.3%)	7 (20.0%)	16 (28.6%)	0.360
**Complication**				
Any	14 (15.4%)	2 (5.7%)	12 (21.4%)	0.043
Infection	14 (15.4%)	2 (5.7%)	12 (21.4%)	0.043
ARDS	9 (9.9%)	2 (5.7%)	7 (19.6%)	0.474
Septic shock	9 (9.9%)	2 (5.7%)	7 (12.5%)	0.474
Cardiac insufficiency	8 (8.8%)	2 (2.9%)	6 (10.7%)	0.706
Metabolic acidosis	8 (8.8%)	1 (2.9%)	7 (12.5%)	0.146
Acute renal injury	5 (5.5%)	1 (2.9%)	4 (7.1%)	0.645
MODS	6 (6.6%)	1 (2.9%)	5 (8.9%)	0.400

Data are *n* (%) unless specified otherwise. *N* is the total number of patients with available data

*P* values for comparing two groups were derived using Fisher’s exact test for categorized variables and the *t*-test for continuous variables

*COVID-19* Coronavirus disease-19; *ARDS* Acute respiratory distress syndrome; *MODS* Multiple organ dysfunction syndrome; *IQR* Interquartile range

### Laboratory examination and imaging findings of patients with COVID-19

**Fig. 2:**
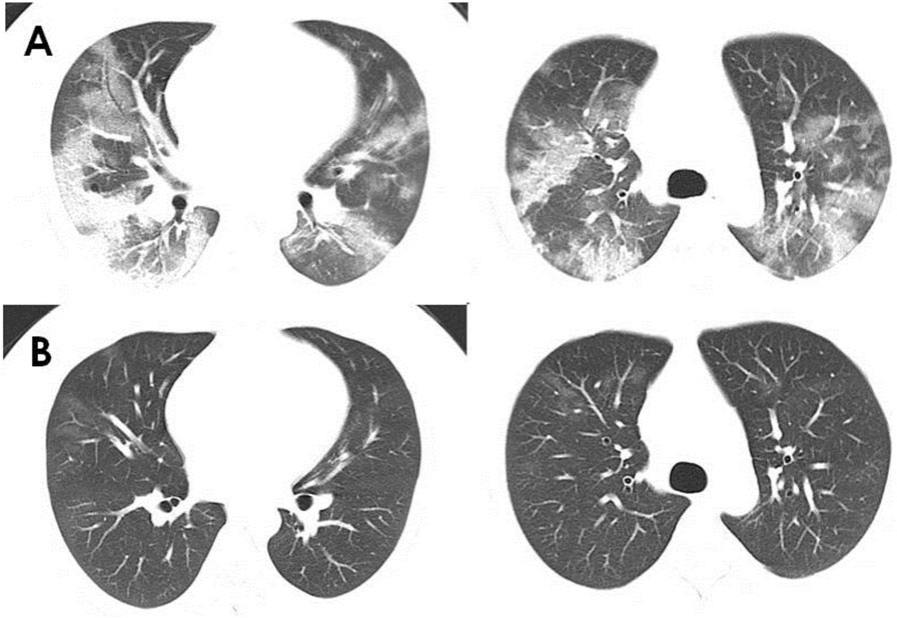
Chest computed tomographic images of a 28-year-old patient with COVID-19. **a**, Chest computed tomographic images obtained on Feb 4, 2020, show ground glass opacity in both lungs on day 5 after symptom onset. **b**, Images taken on Mar 4, 2020 show the absorption of bilateral ground glass opacity after the treatment. COVID-19: Coronavirus disease 2019.

Of the 91 patients, 21 (23.1%) had a white cell count of less than 4.0 ×  10^9^/L, and 39 (42.9%) had a lymphocyte count of less than 1.1 × 10^9^/L. The blood lymphocyte count, platelet count and serum albumin of the imported cases were significantly lower, while the levels of blood creatine kinase and C-reactive protein (CRP) were significantly higher than those in local patients (all *P* <  0.05). All the patients had abnormality in chest CT scans, 79 (86.8%) patients’ lesions were located at the lung periphery, and 75 (82.4%) showed bilateral involvement. The main manifestations were ground-glass opacity (87.9%) and multiple infiltration (83.5%) (Fig. 2). No different imaging features were shown between imported and local patients (Table 3).

**Table 3:** Laboratory data and imaging findings of patients with COVID-19

	All patients (***N*** = 91)	Local cases (***n*** = 35)	Imported cases (***n*** = 56)	***P*** values
**Blood routine**				
White cell count (× 10^9^/L), Mean (*SD*)	5.45 (2.42)	5.57 (1.70)	5.38 (2.79)	0.502
**>** 10.0	5 (5.5%)	1 (2.9%)	4 (7.1%)	0.645
< 4.0	21 (23.1%)	5 (14.3%)	16 (28.6%)	0.116
Lymphocytes (× 10^9^/L), Mean (*SD*)	1.26 (0.62)	1.45 (0.59)	1.14 (0.61)	0.022
< 1.1	39 (42.9%)	8 (22.9%)	31 (55.4%)	0.002
Platelets (× 10^9^/L), Mean (*SD*)	205.8 (69.9)	239.7 (68.3)	184.6 (62.6)	0.001
< 150	23 (25.3%)	3 (8.3%)	20 (35.7%)	0.005
Hemoglobin (g/L), Mean (*SD*)	133.0 (18.1)	131.7 (17.7)	133.8 (18.5)	0.412
**Blood biochemistry**				
Alanine aminotransferase (U/L, normal range 3–35), Median (IQR)	21.3 (14.7–34.8)	22.0 (15.0–31.0)	20.7 (13.8–35.4)	0.427
Increased (*n*, %)	20 (22.0%)	6 (17.1%)	14 (25.0%)	0.379
Aspartate aminotransferase (U/L, normal range 3–40), Median (IQR)	22.0 (17.0–30.1)	24.0 (20.0–32.0)	20.0 (15.3–28.0)	0.170
Increased (*n*, %)	12 (13.2%)	4 (11.4%)	8 (14.3%)	0.761
Total bilirubin (μmol/L, normal range 4.0–17.1), Median (IQR)	9.0 (6.5–13.4)	9.8 (5.9–13.9)	8.4 (6.5–11.8)	0.410
Increased (*n*, %)	13 (14.3%)	3 (8.6%)	10 (17.9%)	0.218
Albumin (g/L, normal range 35.0–55.0), Median (IQR)	42.3 (36.2–47.1)	46.7 (41.4–49.6)	39.3 (34.7–44.4)	0.001
Decreased (*n*, %)	18 (19.8%)	4 (11.4%)	14 (25.0%)	0.114
PT (sec), Median (IQR)	11.3 (10.8–11.8)	11.3 (10.3–12.4)	11.4 (10.8–12.5)	0.407
Serum creatinine (μmol/L), Median (IQR)	65.8 (48.0–76.9)	68.0 (50.9–77.0)	59.0 (47.0–76.8)	0.835
Creatine kinase (U/L), Median (IQR)	63.0 (46.0–93.6)	70.0 (46.0–127.0)	62.0 (45.0–84.0)	0.013
C-reaction protein (mg/L), Median (IQR)	12.3 (2.2–45.1)	5.3 (1.2–30.5)	17.0 (3.0–51.3)	0.022
Procalcitonin (ng/mL), Median (IQR)	0.04 (0.02–0.06)	0.04 (0.02–0.09)	0.03 (0.01–0.06)	0.778
**Chest CT finding**				
Unilateral pneumonia	16 (17.6%)	8 (22.9%)	8 (14.3%)	0.296
Bilateral pneumonia	75 (82.4%)	27 (77.1%)	48 (85.7%)	0.296
Lung periphery	79 (86.8%)	29 (82.9%)	50 (89.3%)	0.526
Ground-glass opacity	80 (87.9%)	30 (85.7%)	50 (89.3%)	0.743
Multiple Infiltration	76 (83.5%)	26 (74.3%)	50 (89.3%)	0.061
Bilateral lung periphery ground-glass opacity	70 (76.9%)	25 (71.4%)	45 (80.4%)	0.325
Nodule	11 (12.1%)	2 (5.7%)	9 (16.1%)	0.193
Lung consolidation	8 (8.8%)	3 (8.6%)	5 (8.9%)	1.000
Pleural effusion	2 (2.2%)	0 (0.0%)	2 (3.6%)	0.521

Data are *n* (%) and mean (SD). *N* is the total number of patients with available data. *P* values for comparing two groups were derived using Fisher’s exact test for categorized variables and the *t*-test for continuous variables

*COVID-19* Coronavirus disease-2019; *CT* Computed tomography; *SD* Standard deviation; *IQR* Interquartile range

### Treatment and prognosis of patients with COVID-19

**Fig. 3:**
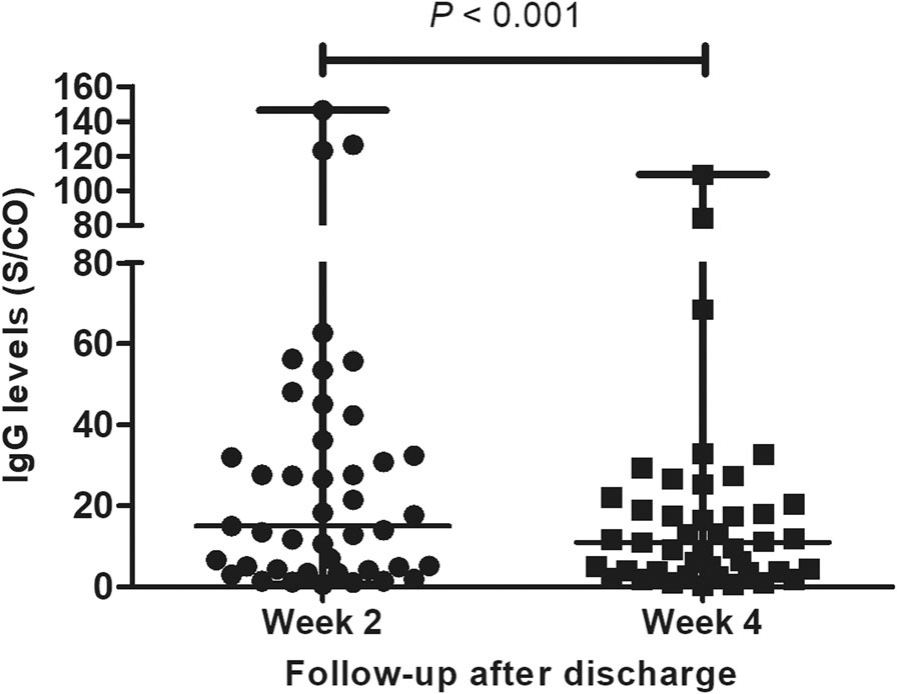
Comparison of the IgG antibody levels of 43 COVID-19 patients between the follow-up of week 2 and week 4 after discharge. COVID-19: Coronavirus disease 2019

The median time that patients stayed in the hospital was 14 days (IQR: 11–18). All the patients took Chinese traditional medicinal treatment, and 89 (97.8%) patients were treated with antiviral therapy, including lopinavir and ritonavir (Kaletra), arbidol and atomized inhalation of interferon α. In total, 22 patients were treated with immunoglobulin, and 20 were treated with thymosin α-1. Overall, 13 patients received short-term corticosteroids treatment, with 40–80 mg/d methylprednisolone for 3–5 days. Only one patient needed extracorporeal membrane oxygenation (ECMO) but died. By March 24, 2020, 88 of 91 patients had been discharged, and three patients had died. There was no significant difference in the treatment, length of hospitalization or clinical outcome between the imported and local patients (Table 4). All the patients had been followed for more than 14 days after discharge, and no nucleic acid detection test for SARS-CoV-2 RNA was returned as positive. IgM and IgG antibodies against SARS-CoV-2 in plasma samples were tested in 83 patients in the follow-up at week 2 after discharge. IgM was positive in 33 patients (39.8%), and IgG was positive in 77 patients (92.8%). As some of the patients had left Hainan, only 43 patients had a second detection of antibodies in the follow-up at week 4 after discharge (median, 48 days from onset; IQR: 44–53 days). Among these patients, 88.4% (38/43) had a reduction in IgG levels. The IgG levels (median S/CO, 10.95; IQR: 3.74–20.95) at week 4 after discharge were significantly lower than the levels (median S/CO, 15.02; IQR: 4.24–36.23) at week 2 (*P* < 0.001, Fig. 3).

**Table 4:** Treatment, virus changes and outcomes of patients with COVID-19

	All patients (***N*** = 91)	Local cases (***n*** = 35)	Imported cases (***n =*** 56)	***P*** value
**Treatment**				
Chinese traditional medicine	91 (100.0%)	35 (100.0%)	56 (100.0%)	1.000
Antiviral therapy	89 (97.8%)	33 (94.3%)	56 (100.0%)	0.112
Oxygen therapy	46 (50.5%)	14 (40.0%)	32 (57.1%)	0.306
Intravenous immunoglobulin therapy	22 (24.2%)	5 (14.3%)	17 (30.4%)	0.081
Thymosin alpha1	20 (22.0%)	9 (25.7%)	11 (19.6%)	0.496
Glucocorticoids	13 (14.3%)	3 (8.6%)	10 (17.9%)	0.218
Intravenous antibiotic	12 (13.2%)	2 (5.7%)	10 (17.9%)	0.120
Mechanical ventilation	10 (11.0%)	2 (5.7%)	8 (14.3%)	0.385
Non-invasive (i.e., face mask)	1 (1.1%)	1 (2.9%)	0 (0.0%)	0.145
Invasive	9 (9.9%)	1 (2.9%)	8 (14.3%)	0.385
ECMO	1 (1.1%)	1 (2.9%)	0 (0.0%)	0.145
Hospital stay, days, Median (IQR)	14 (11–18)	14 (11–17)	15 (11–14)	0.403
**Duration of SARS-CoV-2 RNA positivity from onset**				
Nasopharyngeal swabs, days	11 (6–16)	8 (5–16)	12 (8–16)	0.084
Median (IQR), [range]	[1–39]	[1–37]	[4–39]	
Feces, days	79/91, 13 (10–19)	29/35, 13 (6–18)	50/56, 13 (10–20)	0.216
Median (IQR), [range]	[1–40]	[1–37]	[4–40]	
**Duration of SARS-CoV-2 shedding from onset**				
Nasopharyngeal swabs, days	16 (13–23)	15 (10–22)	17 (14–23)	0.148
Median (IQR), [range]	[6–43]	[8–37]	[6–43]	
Feces (days)	79/91, 19 (14–26)	29/35, 18 (11–24)	50/56, 19 (15–27)	0.242
Median (IQR), [range]	[6–43]	[6–37]	[8–43]	
**Clinical outcome**				
Discharged	88 (96.7%)	34 (97.1%)	54 (96.4%)	1.000
Died	3 (3.3%)	1 (1.1%)	2 (3.6%)	1.000

Data are *n* (%) and mean (SD). *N* is the total number of patients with available data. *P* values for comparing two groups were derived using Fisher’s exact test for categorized variables and the *t*-test for continuous variables

*COVID-19* Coronavirus disease-2019; *SARS-CoV-2* Severe acute respiratory syndrome coronavirus 2; *ECMO* Extracorporeal membrane oxygenation; *NA* Not available; *SD* Standard deviation; *IQR* Interquartile range

### SARS-CoV-2 RNA in nasopharyngeal swabs and feces

We tested for SARS-CoV-2 RNA in nasopharyngeal swabs and feces of all the patients at an interval of 2 to 7 days dynamically. All 91 patients had detectable SARS-CoV-2 RNA in nasopharyngeal swabs, and 79 had detectable levels in feces. SARS-CoV-2 RNA could be detected in nasopharyngeal swabs and feces at medians of 11 days (IQR: 6–16, range: 1–39) and 13 days (IQR: 10–19, range: 1–40), respectively. The viral shedding durations in these two types of samples were 16 days (IQR: 13–23, range: 6–43 days) and 19 days (IQR: 14–26, range: 6–43) from onset, respectively. The durations of SARS-CoV-2 RNA testing being positive and viral shedding in feces were longer than that of nasopharyngeal swabs (*P* = 0.02 and *P* = 0.007, respectively). In samples (including nasopharyngeal swabs and feces) from three dead patients, the SARS-CoV-2 RNA remained positive on the day of death (37 days, 20 days and 17 days from symptom onset, respectively). There was no significant difference in the duration of SARS-CoV-2 RNA positivity or the duration of viral shedding in nasopharyngeal swabs and feces between the imported and local patients (Table 4).

## Discussion

According to the data released by the Hainan government [11], by March 24, 2020, 162 of 168 patients had been discharged and six patients had died, with a mortality rate of 3.6%. No new local cases occurred after February 19, 2020. The 91 cases in this study accounted for 54.2% of all the confirmed cases in Hainan, and a similar trend was found in the epidemic course (see Fig. 1). Of the 91 patients, three patients died. The mortality rate was similar to that of the whole Hainan Province.

Our study shows that during the 29-day epidemic period, most of the patients were diagnosed within the first three weeks after the first identified imported case. In the early period, imported cases were predominant in the epidemic. In the later period, local cases were more common, and 77.1% of the patients showed clustering, mainly in families. Cluster outbreaks were also found in Guangzhou patients in Lin’s study [18]. These findings suggest increasing transmissibility of the virus during the spread [19] and, hence, a great challenge in overall prevention and control. However, the local cases did not lead to continuous community transmission, as reflected by the short epidemic period (29 days). This may be attributed to the strict isolation and prevention measures implemented throughout the country and the effective implementation of prevention and control policies by Hainan (Fig. 1). The measures included establishing fever clinics for screening suspicious patients, designating hospitals to focus on treating patients with COVID-19 [20], and raising the level of emergency response to COVID-19 prevention and control to the first level promptly at day 4. At the same time, other measures also worked well in blocking the routes of transmission and reducing the chance of infection. Examples include encouraging the public to wear face masks, wash hands more frequently, and stay at home unless necessary and activating joint prevention and control mechanisms and cross-sector control for traffic control at the community level. Moreover, delaying the resumption of work and school and implementing work-from-home policies for employees and online teaching for students were adopted to reduce the probability of clusters [21]. The reported estimated incubation time of SARS-CoV-2 was based on limited data. Zhong reported that the median incubation period was four days in 291 cases in China [22]. Xu et al. found that there is no observable difference between the incubation time for SARS-CoV-2, severe acute respiratory syndrome coronavirus (SARS-CoV), and middle east respiratory syndrome coronavirus (MERS-CoV), with a mean of 4.9 days for SARS-CoV-2, 4.7 days for SARS, and 5.8 days for MERS [23]. To avoid the risk of virus spreading, all potentially exposed subjects were required to be isolated for 14 days, which is the longest predicted incubation time. Our epidemiological investigation of 53 patients from Wuhan found that the median time of symptom onset was five days, with a range of one day to 34 days. The patient with the longest incubation period, who was a male in their 70s, flew from Wuhan to Hainan on January 2, 2020 and had no contact with confirmed or suspected COVID-19 patients. He occasionally went to the farmers’ market near his residence to buy vegetables, but there were no confirmed COVID-19 patients associated with the market. He developed symptoms on February 5 and was diagnosed on February 7, 2020 [11]. This particular case indicates that the longest incubation time may be more than 34 days.

This study showed that the main symptoms of patients in Hainan Province were fever and cough, and 30% of patients had shortness of breath. Compared with early COVID-19 cases in Wuhan, diarrhea (14.3%) was relatively more common in Hainan patients [24]. The main complications included infections and ARDS. Six severe cases developed to MODS. In general, the proportions of severe patients and mortalities were lower than those of Wuhan and similar to the national data [25, 26].

In the imported cases, the proportion of patients with fever, the peak temperature, the level of blood CRP, the proportion of severe cases, and the incidence of complications, especially infections, were higher than those in the local cases. Meanwhile, the lymphocyte and platelets counts were significantly lower in imported cases than in local cases. Data showed that the imported cases were older, and coexisting illness was more common than in local cases, which might demonstrate why the imported cases were more severe. Another possible explanation is that the time of infection SARA-CoV-2 in imported cases was earlier, with a more virulent virus subtype. However, this requires further study of the genomics and pathogenicity of SARA-CoV-2 at different stages of transmission. Tang’s research indicates that SARS-CoV-2 had formed two subtypes, S and L, during the transmission process, and changes in viral genes will cause changes in pathogenicity and transmission [19]. A similar study had been conducted for the MERS virus, which had shown that the virus becomes weaker during transmission [27]. It remains to be further studied whether there was virus mutation in the process of virus transmission from imported cases to local cases, which may have led to the weakening of its pathogenicity. Furthermore, given the experience in Wuhan, the Hainan government was well-prepared for the epidemic, and comprehensive screening allowed early case identification and prompt treatment.

All 91 patients, including four asymptomatic patients, had CT changes in the lungs, which mainly manifested as ground-glass opacity in the lung periphery in the early stage. However, as the disease progressed, some patients had pulmonary consolidation and pleural effusion. Therefore, pulmonary CT examination is a sensitive indicator for the screening of COVID-19 and is recommended for all suspected patients [28].

Even so, SARS-CoV-2 RNA provides direct evidence for confirming COVID-19. Among all our patients, SARS-COV-2 RNA was detected in nasopharyngeal swabs, but RNA was not detected in 12 patients’ feces. Due to the positive detecting of SARS-CoV-2 RNA in feces, the problem of gastrointestinal transmission and even aerosol transmission has attracted broad attention. Since then, multiple research teams have isolated viruses in the feces, further illustrating the risk of gastrointestinal transmission.

However, for a new viral infectious disease, there is no exact data on how long virus will be shed through the respiratory and digestive tracts. Our study found that the median duration of fecal SARS-CoV-2 shedding was longer than that in nasopharyngeal swabs, with durations of 19 and 16 days, respectively. And the longest times of SARS-CoV-2 RNA persistent positive testing and viral shedding were 40 days and 43 days, respectively. The relatively long virus shedding duration could pose a great challenge for health systems as the patient pool flows slowly and takes up substantial health facility resources. While it is impossible to host all positive cases in hospitals throughout the virus shedding period, it is possible to shift less-acute cases to other temporary facilities as done in Wuhan. It is worth noting that the nasopharyngeal swabs and feces collected on the day of death of the three critically ill patients were still positive. This suggests that the persistence of the virus may have an impact on the disease prognosis, and it is urgent to screen and develop effective antiviral drugs.

Unfortunately, currently, there are no effective antiviral drugs. Drugs such as remdesivir, kaletra, arbidol, chloroquine phosphate and some Chinese traditional medicines have shown certain effects but still lack rigorous and proven evidence [29–32]. Clinical trials of these drugs are currently ongoing. The treatment of all our patients was basically based on interferon alpha nebulization plus the antiviral regimen of arbidol or kaletra. However, without a controlled study, it is difficult to determine whether it is the natural fluctuation of the virus replication or the effect of the drug.

Since there were no available testing kits in the early stage of the COVID-19 epidemic, data on antibodies against SARS-CoV-2 were only collected in the follow-up after discharge. We observed a high positive rate of IgG and a reduction in the IgG level at a median of 48 days (IQR: 44–53) from symptom onset, which was within six to eight weeks from onset. As reported by Long, IgG levels start to decrease within 2–3 months after infection [33]. These findings may challenge attempts to control COVID-19 through universal immunization, as patients with reduced antibody levels may be re-infected. Of course, the subsequent changes in antibody levels require further observation.

There are some limitations in this study. Due to the barriers to data collection, the clinical data of all 168 patients in the entire Hainan Province have not been collected. In addition, some patients could not be followed up for a long time because they left Hainan after recovery.

## Conclusions

Our study revealed the epidemiological and clinical characteristics and outcomes of imported and local cases outside Hubei Province, suggesting that imported cases were more severe than local cases, but the prognoses could be similarly good. The short epidemic period in Hainan suggests that the epidemic could be quickly brought under control if proper timely measures were taken. This study also suggests that the longest incubation period for COVID-19 may be over 34 days and that the maximum duration of SARS-CoV-2 shedding is at least 43 days. The positive rate of SARS-CoV-2 IgG antibodies was high during convalescence; however, the level of IgG declined from week six to week eight after onset.

## Data Availability

With the permission of the corresponding authors, we can provide participant data without names and identifiers but not the study protocol, statistical analysis plan, or informed consent form. Data can be provided after the Article is published. Once the data can be made public, the research team will provide an email address for communication. The corresponding authors have the right to decide whether to share the data based on the research objectives and plan provided.

## Abbreviations

COVID-19: Coronavirus disease 2019
SARS-CoV-2: Severe acute respiratory syndrome coronavirus 2
S/CO: S/Cut Off
ARDS: Acute respiratory distress syndrome
CT: Computed tomography
CRP: C-reactive protein
PCT: Procalcitonin
CDC: Centre for Disease Control and Prevention
RT-PCR: Reverse transcription polymerase chain reaction
SD: Standard deviation
IQR: Interquartile range
MODS: Multiple organ dysfunction syndrome
ECMO: Extracorporeal membrane oxygenation

## References

[1] Zhou P, Yang XL, Wang XG, Hu B, Zhang L, Zhang W, et al. A pneumonia outbreak associated with a new coronavirus of probable bat origin. Nature. 2020 Feb 3. 10.1038/s41586-020-2012-7. 10.1038/s41586-020-2012-7 PMC7095418 32015507

[2] Zhu N, Zhang D, Wang W, Li X, Yang B, Song J (2020). A novel coronavirus from patients with pneumonia in China, 2019. N Engl J Med.

[3] WHO Coronavirus Disease (COVID-19) Dashboard. https://covid19.who.int/ Accessed 7 Sept 2020.

[4] Huang C, Wang Y, Li X, Ren L, Zhao J, Hu Y (2020). Clinical features of patients infected with 2019 novel coronavirus in Wuhan, China. Lancet.

[5] Li X, Zai J, Wang X, Li Y (2020). Potential of large "first generation" human-to-human transmission of 2019-nCoV. J Med Virol.

[6] Liu Y, Gayle AA, Wilder-Smith A, Rocklöv J. The reproductive number of COVID-19 is higher compared to SARS coronavirus. J Travel Med. 2020. 10.1093/jtm/taaa021. 10.1093/jtm/taaa021 PMC7074654 32052846

[7] Zhao S, Musa SS, Lin Q, Ran J, Yang G, Wang W (2020). Estimating the Unreported Number of Novel Coronavirus (2019-nCoV) Cases in China in the First Half of January 2020: A Data-Driven Modelling Analysis of the Early Outbreak. J Clin Med.

[8] Xu K, Cai H, Shen Y, Ni Q, Chen Y, Hu S (2020). Management of corona virus disease-19 (COVID-19): the Zhejiang experience. Zhejiang Da Xue Xue Bao Yi Xue Ban.

[9] Xia J, Tong J, Liu M, Shen Y, Guo D. Evaluation of coronavirus in tears and conjunctival secretions of patients with SARS-CoV-2 infection. J Med Virol. 2020. 10.1002/jmv.25725. 10.1002/jmv.25725 PMC7228294 32100876

[10] http://www.comrc.com.cn/news/1435.html. Accessed 8 Sept 2020.

[11] http://wst.hainan.gov.cn/yqfk/. Accessed 8 Sept 2020.

[12] World Health Organization. Clinical management of severe acute respiratory infection when novel coronavirus (nCoV) infections suspected: interim guidance. Published January 28, 2020. Accessed 7 Sept 2020.

[13] Chan JF, Yuan S, Kok KH, Chu H, Yang J, To KK (2020). A familial cluster of pneumonia associated with the 2019 novel coronavirus indicating person-to-person transmission: a study of a family cluster. Lancet..

[14] Jin YH, Cai L, Cheng ZS, Cheng H, Deng T, Fan YP (2020). A rapid advice guideline for the diagnosis and treatment of 2019 novel coronavirus (2019-nCoV) infected pneumonia (standard version). Mil Med Res.

[15] Fan E, Brodie D, Slutsky AS (2018). Acute Respiratory Distress Syndrome: Advances in Diagnosis and Treatment. JAMA.

[16] Metlay JP, Waterer GW, Long AC, Anzueto A, Brozek J, Crothers K (2019). Diagnosis and treatment of adults with community-acquired pneumonia. An official clinical practice guideline of the American Thoracic Society and Infectious Diseases Society of America. Am J Respir Crit Care Med.

[17] https://www.who.int/publications/i/item/laboratory-testing-of-2019-novel-coronavirus-(-2019-ncov)-in-suspected-human-cases-interim-guidance-17-january-2020. Accessed 08 Sept 2020.

[18] Ziying L, Huijuan C, Yusheng J, Zhanlian H, Guo X, Junfeng C (2020). A cross-sectional comparison of epidemiological and clinical features of patients with coronavirus disease (COVID-19) in Wuhan and outside Wuhan, China. Travel Med Infect Dis.

[19] Tang X, Wu C, Li X, Song Y, Yao X, Wu X et al. On the origin and continuing evolution of SARS-CoV-2. *National Science Review*, nwaa036, 10.1093/nsr/nwaa036. Accessed 7 Sept 2020. 10.1093/nsr/nwaa036 PMC7107875 34676127

[20] http://wst.hainan.gov.cn/swjw/rdzt/yqfk/202001/t20200131_2742298.html Accessed 7 Sept 2020.

[21] http://www.gov.cn/zhengce/content/2020-01/27/content_5472352.html. Accessed 7 Sept 2020.

[22] Guan WJ, Ni ZY, Hu Y, Liang WH, Ou CQ, He JX, et al. Clinical characteristics of coronavirus disease 2019 in China. N Engl J Med. 2020. 10.1056/NEJMoa2002032. 10.1056/NEJMoa2002032 PMC7092819 32109013

[23] Jiang X, Rayner S, Luo MH. Does SARS-CoV-2 has a long incubation period than SARS and MERS? J Med Virol. 2020. 10.1002/jmv.25708. 10.1002/jmv.25708 PMC7166592 32056235

[24] Chen N, Zhou M, Dong X, Qu J, Gong F, Han Y (2020). Epidemiological and clinical characteristics of 99 cases of 2019 novel coronavirus pneumonia in Wuhan, China: a descriptive study. Lancet..

[25] Wang D, Hu B, Hu C, Zhu F, Liu X, Zhang J, et al. Clinical characteristics of 138 hospitalized patients with 2019 novel coronavirus-infected pneumonia in Wuhan, China. JAMA. 2020. 10.1001/jama.2020.1585. 10.1001/jama.2020.1585 PMC7042881 32031570

[26] Xu XW, Wu XX, Jiang XG, Xu KJ, Ying LJ, Ma CL (2020). Clinical findings in a group of patients infected with the 2019 novel coronavirus (SARS-Cov-2) outside of Wuhan, China: retrospective case series. BMJ.

[27] Kim KH, Tandi TE, Choi JW, Moon JM, Kim MS (2017). Middle East respiratory syndrome coronavirus (MERS-CoV) outbreak in South Korea, 2015: epidemiology, characteristics and public health implications. J Hosp Infect.

[28] Xie X, Zhong Z, Zhao W, Zheng C, Wang F, Liu J (2020). Chest CT for typical 2019-nCoV pneumonia: relationship to negative RT-PCR testing. Radiology..

[29] Gao J, Tian Z, Yang X. Breakthrough: Chloroquine phosphate has shown apparent efficacy in treatment of COVID-19 associated pneumonia in clinical studies. Biosci Trends. 2020 Feb 19. 10.5582/bst.2020.01047. 10.5582/bst.2020.01047 32074550

[30] Zhang Q, Wang Y, Qi C, Shen L, Li J. Clinical trial analysis of 2019-nCoV therapy registered in China. J Med Virol. 2020 Feb 28. 10.1002/jmv.25733. 10.1002/jmv.25733 PMC7228274 32108352

[31] Li H, Wang YM, Xu JY, Cao B (2020). Potential antiviral therapeutics for 2019 novel coronavirus. Zhong Hua Jie He He Hu Xi Za Zhi.

[32] Wang M, Cao R, Zhang L, Yang X, Liu J, Xu M (2020). Remdesivir and chloroquine effectively inhibit the recently emerged novel coronavirus (2019-nCoV) in vitro. Cell Res.

[33] Long QX, Tang XJ, Shi QL, Li Q, Deng HJ, Yuan J, et al. Clinical and immunological assessment of asymptomatic SARS-CoV-2 infections. Nat Med. 2020. 10.1038/s41591-020-0965-6. 10.1038/s41591-020-0965-6 32555424

